# Centrifuge-Free Bone Marrow Aspirate Harvest for Orthopaedic Applications

**DOI:** 10.1016/j.eats.2025.103721

**Published:** 2025-07-02

**Authors:** Sarah Oyadomari, Anil Ranawat, Dean Wang

**Affiliations:** a Department of Orthopaedic Surgery, University of California, Irvine, Irvine, California, U.S.A.; b Hospital for Special Surgery, New York, New York, U.S.A.; c Department of Biomedical Engineering, University of Irvine, Irvine, California, U.S.A.

## Abstract

Autologous bone marrow aspirate (BMA) is increasingly being used in clinical practice to augment the repair and healing of bone, cartilage, and tendon by delivering mesenchymal stem cells (MSCs) and growth factors to the site of injury. Traditional BMA harvest and preparation rely on centrifugation to generate a BMA concentrate with a higher concentration of MSCs than the original aspirate. However, this traditional technique may be inefficient and time-consuming, and it could sacrifice sterility. In this technique, we describe a centrifuge-free BMA harvest system (Marrow Cellution; Cervos Medical) that avoids peripheral blood dilution during aspiration, thereby potentially achieving a higher yield of MSCs and growth factors within the BMA without the need for centrifugation.

Application of autologous bone marrow aspirate (BMA) to treat various orthopaedic pathologies, including tendinopathy and osteoarthritis, has shown early promising results.^1^, ^2^, ^3^, ^4^ The benefits of BMA treatment, beyond augmentation of bone healing, are thought to be derived from mesenchymal stem cells (MSCs) within the bone marrow as well as growth factors and cytokines that facilitate a favorable biologic environment for musculoskeletal tissue healing and homeostasis.^5^^,^^6^ Although BMA can be harvested from various sites, aspiration from the iliac crest has shown to result in the highest concentration of MSCs compared to aspiration from other sites such as the distal femur.^7^, ^8^, ^9^

Traditional preparation of BMA for orthopaedic applications involves harvest of a large volume of bone marrow aspirate from a single location within cancellous bone, then filtering and centrifuging the aspirate to produce a higher concentration of MSCs.^10^^,^^11^ However, this process to form BMA concentrate (BMAC) has several disadvantages, including peripheral blood dilution during harvest, waste of valuable cytokines during filtration and centrifugation, time needed for centrifugation, and sterility concerns. Traditional commercial BMA cannulas feature both an open distal end and side fenestrations (Fig 1). With these traditional cannulas, the syringe suction pressure primarily favors the larger open distal end over the smaller side fenestrations, and studies have shown that aspiration after the first 1 to 2 mL is typically diluted with peripheral blood rather than the desired bone marrow containing MSCs.^8^, ^9^, ^10^, ^11^, ^12^, ^13^, ^14^, ^15^ Filtration and centrifugation counteract this dilution; however, the supernatant that is discarded may be rich in other beneficial cells and growth factors. Moreover, centrifugation requires breaking the sterile surgical field, introducing opportunities for not only contamination but also potential errors with further handling and increased preparation time. A centrifuge-free BMA system (Marrow Cellution; Cervos Medical) overcomes the current shortcomings of traditional BMAC systems while maximizing the MSC yield within BMA, preserving sterility, and increasing efficiency.^16^

**Fig 1:**
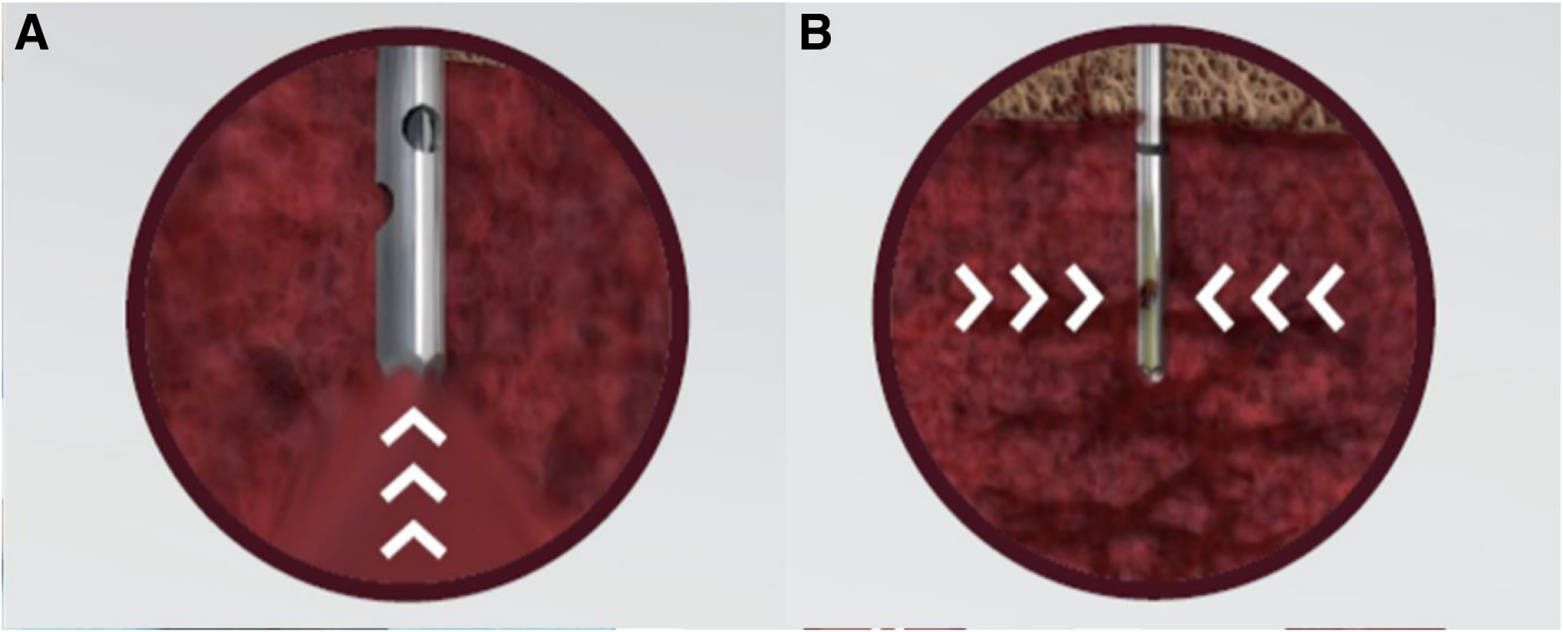
Cannula design. (A) Traditional bone marrow aspirate needle with open distal tip. (B) The Marrow Cellution (Cervos Medical) needle with a closed distal tip after placement of the aspiration cannula. Aspiration through the side fenestrations ensures bone marrow aspirate is being drawn from different regions of the endosteal bone and prevents peripheral blood dilution through the larger distal lumen.

## Surgical Technique

### Preparation

**Fig 2:**
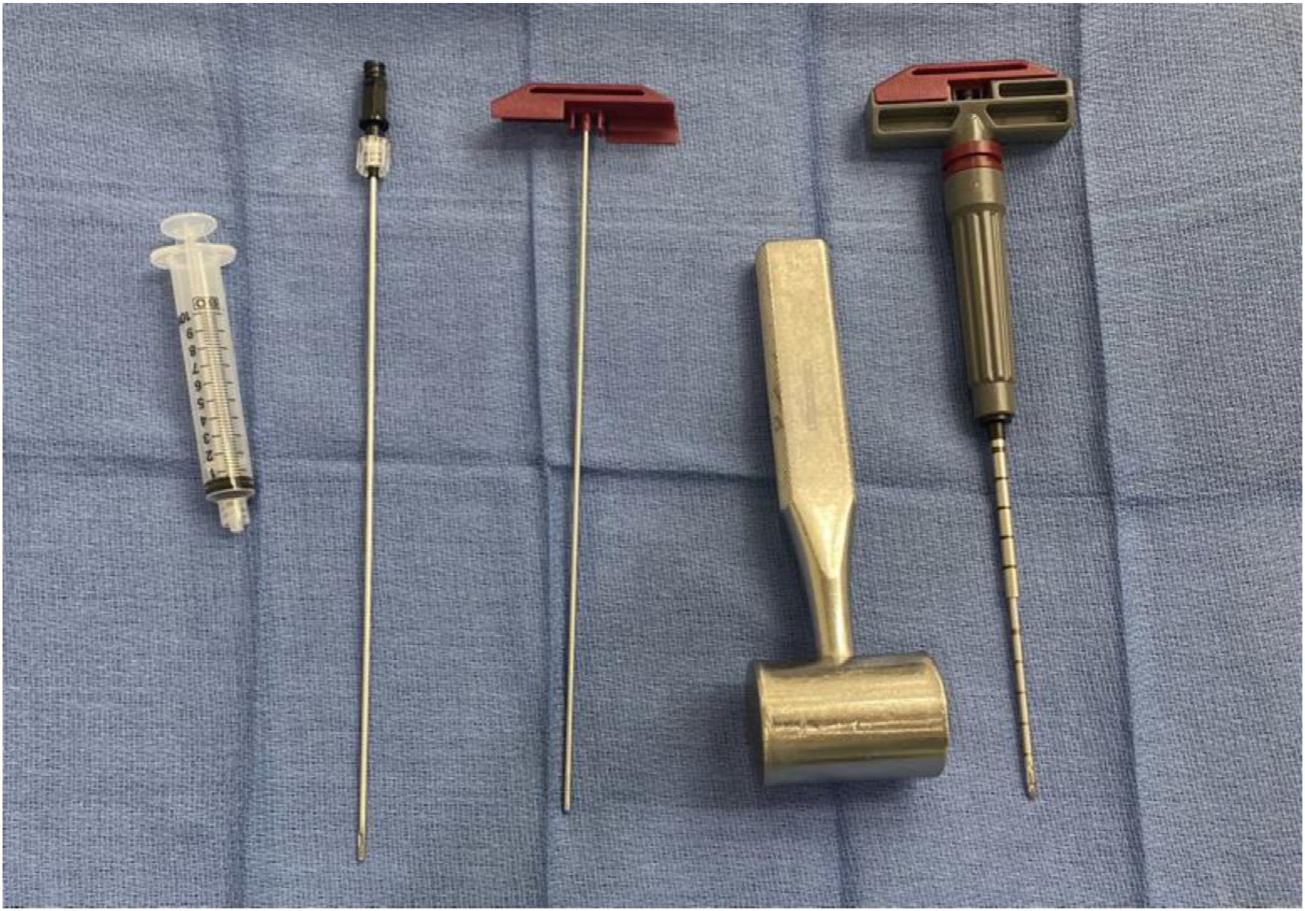
Centrifuge-free bone marrow aspirate harvest sterile setup (Marrow Cellution; Cervos Medical). Pictured left to right are (1) a 10-mL syringe, (2) an aspiration cannula, (3) a blunt introducer stylet, (4) a mallet, and (5) a sharp introducer stylet assembled into the access cannula.

The BMA harvest is performed after anesthesia is administered to the patient and before extremity prep and surgical draping. A sterile Mayo stand is prepared with the contents of the BMA kit: access cannula, introducer stylet (blunt and sharp), aspiration cannula, and a 10-mL syringe (Fig 2). Heparin is mixed with sterile saline to create a final heparin solution with a concentration of 2,000 U/mL. All cannulas, syringes, and anything that will be used to store the BMA (e.g., specimen cups) should be flushed and rinsed with the heparin solution to prevent clotting of the BMA.

The harvest site over the anterior iliac crest is cleaned with chlorhexidine, and a miniature sterile field is created using a towel with a cutout in the center placed over the iliac crest.

### Insertion of the Cannula

The proper harvest site is identified by palpating the anterior superior iliac spine and moving cephalad along the crest 3 to 4 cm. The sharp stylet is placed inside the access cannula, and the device is manually punctured through the skin and subcutaneous layer. The distal end of the cannula is used to feel the inner and outer tables of the iliac crest and then centered on the anterior cortex. It is then malleted through the cortex of the iliac crest and into the medullary space. The sharp stylet is then exchanged for the blunt stylet, and the cannula is advanced with a mallet, following the plane of the iliac crest and staying between the inner and outer tables, until the outer cannula of the access needle rests on the cortical bone.

### Bone Marrow Aspiration

Once at the appropriate level, the blunt stylet is removed and exchanged for the aspiration cannula, which closes the distal lumen of the needle while keeping the side fenestrations open. A 10-mL syringe is attached to the end of the aspirator cannula. The plunger on the syringe is pulled back rapidly in short bursts to aspirate 0.5 to 1 mL of bone marrow from a single intramedullary location. Next, while holding the outer housing in place, the handle is rotated counterclockwise 360° with the opposite hand to raise the access needle tip to a new intramedullary location (Video 1). This “snapback” aspiration process is repeated until the desired amount of BMA is achieved. The BMA is then stored in a specimen cup on the sterile back table.

### Removal of Cannula

The aspiration cannula is removed, and the blunt stylet is placed back into the access cannula. The entire device is removed from the iliac crest by simultaneously turning and withdrawing the cannula. The puncture wound is then dressed with gauze and Tegaderm (3M).

## Discussion

**Fig 3:**
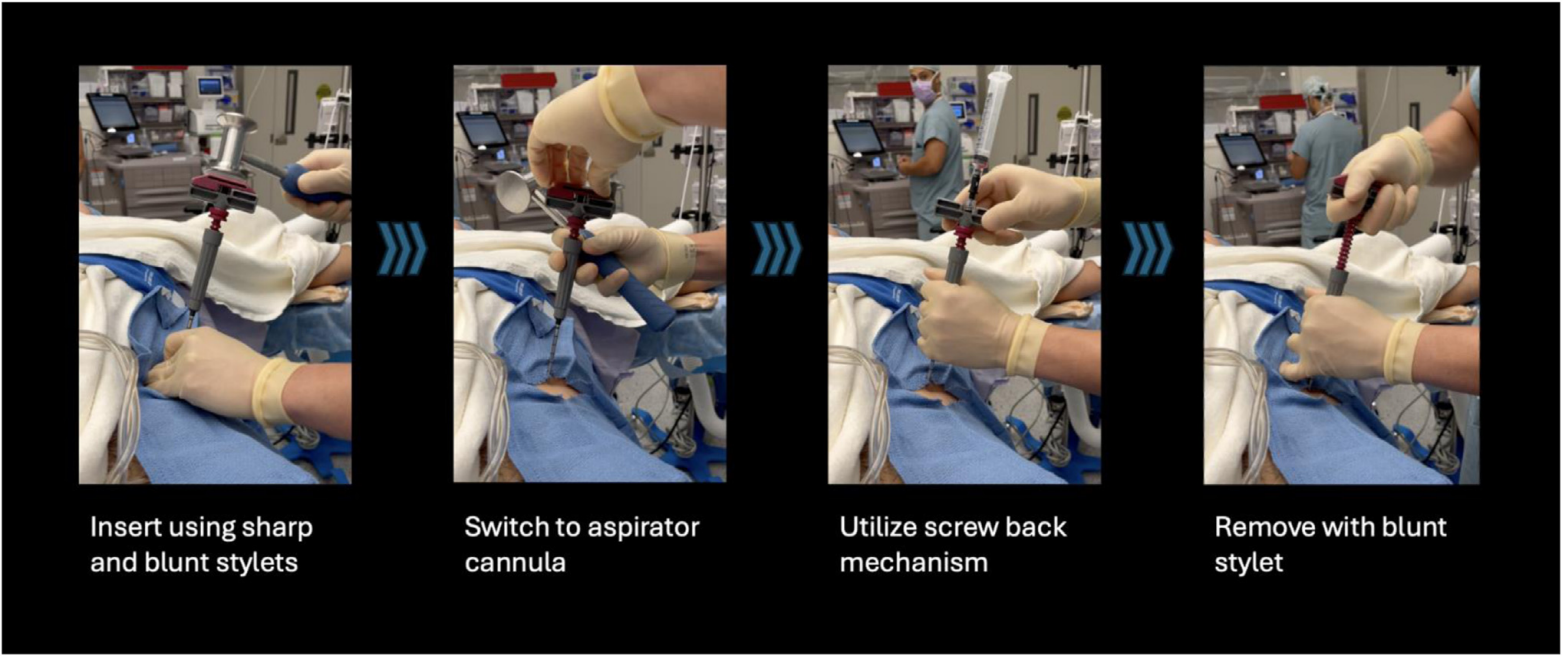
Overview of steps for centrifuge-free bone marrow aspirate harvest. The patient is supine on the operating table, and the left iliac crest is prepped and draped with a blue towel. All pictures are from a viewpoint looking cranially toward the head of the patient. Steps of the harvest: (1) insertion of the access cannula, (2) switching the stylet to the aspiration cannula, (3) “snapback” harvest of bone marrow aspirate through the aspiration cannula and utilizing the screw-back mechanism to harvest from multiple areas within the intramedullary space, and (4) removal of the device, pictured from left to right.

To maximize MSC yields with traditional aspiration cannulas, multiple cortical perforations with repositioning of the cannula and drawing small volumes of bone marrow from multiple geographies within the medullary space are needed to prevent peripheral blood dilution.^2^^,^^15^ This system improves the efficiency of BMA harvest during aspiration by closing the larger distal end of the aspiration cannula and using the side fenestrations only to draw bone marrow from the cell-rich endosteal surfaces (Table 1). Additionally, the screw-back mechanism easily repositions the cannula in discrete increments to draw from new geographies within the intramedullary marrow space, and the 10-mL syringe allows for short pulses of negative pressure to draw 0.5 to 1 mL of bone marrow at a time. Utilizing this process, the system eliminates the need for centrifugation by preventing peripheral blood dilution and avoids the use of a filter, potentially resulting in a higher yield of MSCs and growth factors than current commercial BMAC systems (Table 2).^16^^,^^17^ Furthermore, this centrifuge-free process can be done in a few minutes in a sterile environment (Fig 3), saving time, excess handling, and possible contamination associated with centrifugation.

**Table 1:** Pearls and Pitfalls for the Centrifuge-Free BMA Harvest Technique With Marrow Cellution (Cervos Medical)

Pearls	Pitfalls
Ensure that you flush all tools that contact the BMA with heparin before BMA harvest	Without a heparin flush, the BMA may clot in the syringe when stored on the back table
During insertion of the cannula, there will be a pitch change when passing the cortex	Using the sharp stylet while in the intramedullary space risks perforating the far cortex
Use a “snapback” aspiration process from multiple endosteal locations to maximize the concentration of MSCs	Aspirating more than 1 to 2 mL from a single location in the intramedullary space causes peripheral blood dilution

BMA, bone marrow aspirate; MSC, mesenchymal stem cell.

**Table 2:** Advantages and Disadvantages of the BMA Harvest Technique With Marrow Cellution (Cervos Medical)

Advantages	Disadvantages
Centrifuge-free technique saves on time	Higher cost of Marrow Cellution needle compared to traditional Jamshidi needles
Centrifuge-free technique avoids off-field processing and sterility concerns	Peripheral blood dilution can still occur when aspirating more than 1 to 2 mL from a single location in the intramedullary space
Aspiration cannula closes off the distal lumen, allowing for BMA from the side fenestrations	The ideal bone marrow solution to augment tendon, ligament, and other musculoskeletal soft tissue healing is still not well understood
Screw-back mechanism allows for easy aspiration from different areas of the intramedullary space	

BMA, bone marrow aspirate.

## Disclosures

The authors declare the following financial interests/personal relationships which may be considered as potential competing interests: A.R. receives funding grants from DePuy Synthes Mitek Sports Medicine; is a consultant or advisor for Anika Therapeutics, Bodycad, Cervos, CONMED, Moximed, Ranfac, and Smith & Nephew; has equity or stocks with Overture Orthopaedics and Enhatch; has a patent with royalties paid to DePuy; and is a board member of American Academy of Orthopaedic Surgeons, American Orthopaedic Society of Sports Medicine, and Eastern Orthopaedic Association. D.W. is a board member of the Western Orthopaedic Association; is a consultant or advisor for Stryker, Cervos, DePuy Synthes Mitek Sports Medicine, and Newclip Technics; receives speaking and lecture fees from Arthrex and Vericel; receives funding grants from Vericel and Immunis; and has equity or stocks with Overture Orthopaedics, Cartilage, and ReliaCare Solutions. AThe other author (S.O.) declares that they have no known competing financial interests or personal relationships that could have appeared to influence the work reported in this paper.

## Supplementary Data

Video 1 Centrifuge-free bone marrow aspirate harvest techniquefor orthopaedic applications. Bone marrow is aspirated from the left iliac crest of a patient in the supine position.
